# The angiotensin converting enzyme insertion/deletion polymorphism alters the response of muscle energy supply lines to exercise

**DOI:** 10.1007/s00421-012-2583-6

**Published:** 2013-02-09

**Authors:** David Vaughan, Felicitas A. Huber-Abel, Franziska Graber, Hans Hoppeler, Martin Flück

**Affiliations:** 1 Institute for Biomedical Research into Human Movement and Health, School of HealthCare Science, Manchester Metropolitan University, Chester Street, Manchester, M1 5GD UK; 3 Department of Orthopaedics, University of Zürich, Balgrist University Hospital, Zurich, Switzerland; 2 Department of Anatomy, University of Berne, Berne, Switzerland

**Keywords:** Vasoconstriction, Metabolism, Exercise, Human, Genetical genomics, Aerobic capacity, Capillary

## Abstract

The presence of a silencing sequence (the I-allele) in the gene for the upstream regulator of blood flow, angiotensin I-converting enzyme (*ACE*), is associated with superior endurance performance and its trainability. We tested in a retrospective study with 36 Caucasian men of Swiss descent whether carriers of the *ACE I*-allele demonstrate a modified adaptive response of energy supply lines in knee extensor muscle, and aerobic fitness, to endurance training based on 6 weeks of supervised bicycle exercise or 6 months of self-regulated running (*p* value <Bonferroni-corrected 5 %). Body weight related maximal oxygen uptake and capillary density in *vastus lateralis* muscle before training were 20 and 23 % lower, respectively, in carriers of the I-allele. Bicycle (*n* = 16) but not running type endurance training (*n* = 19) increased the volume content of subsarcolemmal mitochondria (2.5-fold) and intramyocellular lipid (2.1-fold). This was specifically amplified in I-allele carriers after 6 weeks of bicycle exercise. The enhanced adjustment in myocellular organelles of aerobic metabolism with bicycle training corresponded to *ACE I*-allele dependent upregulation of 23 muscle transcripts during recovery from the bicycle stimulus and with training. The majority of affected transcripts were associated with glucose (i.e. ALDOC, Glut2, LDHC) and lipid metabolism (i.e. ACADL, CPTI, CPTII, LIPE, LPL, FATP, CD36/FAT); all demonstrating an enhanced magnitude of change in carriers of the *ACE I*-allele. Our observations suggest that local improvements in mitochondrial metabolism, through a novel expression pathway, contribute to the varying trainability in endurance performance between subjects with genetically modified expression of the regulator of vascular tone, ACE.

## Introduction

Variability in the response of aerobic capacity (*V*O_2max_) to endurance training is a common observation in human experimentation (Bouchard and Rankinen 2001; Timmons 2011). Pre-training status and familial factors such as shared environment and genetics have been proposed to explain inter-subject differences in the response to regular physical activity (Bouchard and Rankinen 2001). A volumetric increase in cellular constituents of energy supply (i.e. capillarity, mitochondria and intramyocellular lipid) in *vastus lateralis* muscle makes an important local contribution to the increase in *V*O_2max_ with endurance training (Hoppeler et al. 1985; Hoppeler and Weibel 1998). Plasticity of energy supply lines in muscles being recruited during leg exercise (Krustrup et al. 2004) likely contributes to inter-individual differences in the response of aerobic metabolism to training.

A number of gene polymorphisms have been identified, which relate to differences in the exercise phenotype and their trainability (Bray et al. 2009). A commonly studied candidate for genetically determined physical performance is the presence of an insertion DNA sequence comprising a silencer region within intron 16 of the *ACE* gene (the I-allele; Myerson et al. 1999), which occurs frequently in Caucasian populations (Lester et al. 1999). The presence of the *ACE I*-allele in *ACE*-*II* and *ACE*-*ID* genotypes (i.e. I-allele carriers) is associated with elevated endurance performance and its trainability compared to homozygotes of the D-allele (i.e. *ACE*-*DD* genotypes) that do not carry the *ACE I*-allele (Jones et al. 2002; Myerson et al. 1999). For instance, Myerson et al. (1999) and Alvarez et al. (2000) report that the frequency of the *ACE I*-allele is increased in subjects with elite endurance performance. The effect of carrying the I-allele is related to lowered expression and activity of the encoded *ACE* enzyme (Almeida et al. 2010; Alvarez et al. 2000; Rigat et al. 1990). This enzyme catalyses the proteolytic conversion of angiotensin 1 into the bioactive vasoconstrictory peptide angiotensin 2 (Ang2), (Munzenmaier and Greene 1996), while accelerating degradation of kinins that act as vasodilators (Dietze and Henriksen 2008; Woods et al. 2002).

The mechanism driving the association of endurance performance with the *ACE I/D*-allele polymorphism with specific emphasis for working muscle is not understood (Baker and Davids 2006). A number of processes downstream of *ACE* activity have been suggested to contribute to elevated trainability of endurance performance in carriers of the *ACE I*-allele (Puthucheary et al. 2011). Potentially, this involves Ang2 and kinin modulated glucose and triglyceride supply and metabolism in working muscle (Dietze and Henriksen 2008; Jamerson et al. 1996) or vascular processes associated with contraction-induced inhibition of Ang2 regulated smooth muscle cell contractility that allows recruitment of new capillaries to perfusion (Brothers et al. 2006). In this regard, Ang2-mediated modification of angiogenesis by Ang2-stimulated endothelial cell proliferation (Amaral et al. 2001; Bellamy et al. 2010; Munzenmaier and Greene 1996) is of particular interest. Indeed, exercise-induced capillary growth is related to increased expression of angiogenic gene transcripts in skeletal muscle post exercise (Yan et al. 2011), and a number of genes have been shown recently to lie downstream of Ang2 signalling (Song et al. 2011). There is evidence that genetically modified *ACE* expression in mice alters muscle capillarity (Zhang et al. 2005) and that the *ACE I/D* polymorphism affects fibre type distribution (Zhang et al. 2003). A scenario is suggested whereby soluble mediators of ACE action reaching the vascular bed of skeletal muscle upon contraction-induced vasodilatation regulate muscle perfusion and drive capillary growth during recovery from exercise.

We reasoned that the effect of the *ACE I/D* polymorphism on aerobic fitness has a muscle component related to exercise-induced capillary growth and substrate supply. Specifically, we hypothesised that *ACE*-*DD* genotypes compared to carriers of the I-allele (II and ID genotypes) would demonstrate an amplified gene response of angiogenic and metabolic pathways in the knee extensor muscle, *vastus lateralis*, and corresponding cellular endpoints of energy supply (i.e. capillarity, lipid and mitochondrial content) to single and repeated endurance exercise during training. Differences in the association between endurance phenotypes and the *ACE I*-allele have been studied in endurance runners and cyclists (Alvarez et al. 2000; Myerson et al. 1999) and have been suggested to be modulated by the type/intensity of endurance training (Cam et al. 2007). We, therefore, tested whether adjustments of energy supply lines in *vastus lateralis* muscle as a function of the *ACE I*-allele would differ between bicycle and running type endurance training.

## Materials and methods

### Experimental design

A retrospective experiment was carried out in two groups of healthy Caucasian men of Swiss descent to characterize skeletal muscle’s response to endurance training in function of the *ACE I*-allele. Therefore, genotyping for the *ACE I*/*D* polymorphism was carried out on samples from *vastus lateralis* muscle that had been subjected to the analysis of muscle structure and aerobic capacity before and after participants completed two types of endurance training based on (1) bicycle (group one) or (2) running (group two) exercise. Transcript profiling was performed on biopsy samples of *vastus lateralis* muscle from the bicycle group being collected prior and over a time course over the first 24 h of recovery from the first endurance exercise test on a stationary bicycle. *M. vastus lateralis* was chosen as the study object due to its important recruitment during the exercise tests on the bicycle and training (Krustrup et al. 2004).

### Subjects

The study characterises 36 healthy Caucasian subjects of Swiss descent. This comprised a first group involving 17 not systematically trained men which underwent endurance training on a stationary bicycle as described (Schmutz et al. 2006, 2010). The second group includes 19 men who underwent running type endurance training as reported (Suter et al. 1995). Age, height and body weight were recorded for all subjects. The investigations were conducted with permission of the Ethics Committee of Bern, Switzerland, in compliance with the Helsinki Convention for Research on human participants.

### Genotyping

DNA isolation was performed from pooled cryosections of approximately 10 mg tissue of *M. vastus lateralis* following a commercially available protocol (Qiagen DNeasy Blood and Tissue Handbook, 07/2006, cat. no. 69504). *ACE I/D* genotyping was carried out with polymerase chain reaction (PCR) as described by Evans et al. (1994) on isolated DNA. The primers corresponded to those established previously for the identification of the *ACE I/D* polymorphism (for details see Genbank number X62855):

Detection of the 83 bp amplicon specific to the absence of the insertion sequence (i.e. the D-allele) was achieved by a combination of *ACE*1 (5′-catcctttctcccatttctc-3′) and *ACE*3 (5′-atttcagagctggaataaaatt-3′) primers. *ACE*2 (5′-tgggattacaggcgtgatacag-3′) and *ACE*3 (5′-atttcagagctggaataaaatt-3′) primers were applied to detect the 66 bp amplicon specific for the I-allele in intron 16 of the *ACE* gene.

PCR reactions were run with a mix of the three primers using Sybr Green master mix (Applied Biosystems) on a Biorad DNA machine controlled by the MJ Opticon Monitor software (Biorad). This involved 45 standard cycles of denaturing at 95 °C for 15 s followed by annealing and extension at 55 °C for 1 min. Amplicon identification followed using a melting curve analysis between a temperature range of 70–80 °C. Identity of the amplified sequence for the *ACE*-*I* and *ACE*-*D* allele was validated by sequencing of the PCR products with the specific primers (Microsynth, Balgach, Switzerland). Presence of the short amplicon for the I-allele was identified by lower melting temperature (73.5 °C) compared to the longer D-allele (75.5 °C). Samples with poor signal to noise ratio were re-run in separate reactions with the specific primer pair for amplification of either the I-allele or D-allele.

### Whole body aerobic capacity

Before and after endurance training, *V*O_2_max in [L O_2_ min^-1^] was determined with ergospirometry in an incremental endurance test on a stationary bicycle ergometer (Jaeger, Ergoline 800S, Bitz, Germany) as described (Schmutz et al. 2006; Suter et al. 1995). The exercise started with 40 W, the workload being increased by 30 W every 2 min until the subjects could no longer maintain a cadence of more than 60 rpm. Criteria for the achievement of *V*O_2max_ were a non-linear increase in *V*O_2_ and lactate levels >7 mM and a respiratory exchange ratio >1.1. Subsequently, body weight related VO_2_max (i.e. relative *V*O_2max_) was calculated and given in the units [mL O_2_ min^-1^, kg^-1^].

### Endurance exercise test

Subjects of group one reported to the laboratory in the morning after an overnight fast. A resting biopsy was collected from *vastus lateralis* muscle in the rested state. On a separate occasion, 57 ± 4 h later, *V*O_2max_ was determined. Two weeks later participants reported to the laboratory after an overnight fast. They carried out a bout of two-legged endurance exercise on the Ergoline 800S ergometer consisting of a warm-up of 10 min at 40 % of PPO (peak power output) followed by 30 min of cycling at 65 % PPO. Oxygen consumption was recorded during the exercise. Muscle biopsies were collected 1, 3, 8 and 24 h after exercise from *vastus lateralis* muscle in alternating order from new incision sites in the left or right leg, frozen in nitrogen-cooled isopentane and stored in liquid nitrogen.

### Endurance training

Subjects enrolled in either of two endurance-training programs. Group one entered a supervised bicycle-training program subsequent to the single bout of exercise. This comprised five controlled 30 min exercise sessions per week on a stationary bicycle ergometer (Kettler, Ense-Parsit, Germany) at 65 % PPO for a total of 6 weeks. Each training session was monitored based on heart rate measures with a chest belt (Accurex Plus, Polar Electro Finland, Kempele, Finland) and adjusted to maintain a constant individual training intensity at approximately 90 % of maximal heart rate. In addition, training workload was followed by Borg’s Perceived Exertion and Pain Scale and weekly measures of lactate during exercise (finger tip, Yellow Springs Lactate Analyzer 23L). At the end of the training period after 3 days of rest, a post training biopsy was collected from *vastus lateralis* muscle. Whole body aerobic capacity was re-assessed 3 days later. One subject dropped out due to an unrelated accident.

Subjects from group two had their whole body aerobic capacity determined 3 weeks before entering a 6-month endurance exercise training program composed of home-based running of attempted 4 × 30 min per week for 6 months at an intensity corresponding to 75 % *V*O_2max_ as described (Suter et al. 1995). Participants were instructed to keep a training diary, containing the heart rate, time and distance covered during each training session. They had to control their heart rate during exercise and recovery with a portable heart rate monitor (Polar Edge, Polar Electro, Kempele, Finland). Subjects were instructed to maintain the heart rate during the running exercise to a value corresponding to 75 % *V*O_2max_ as determined in the lab-based test. Only subjects meeting the criteria of an average training duration of 60 min per week were included in this analysis. The average training activity over the 6 months training period was 105 min and 18.8 km per week. After endurance training, whole body aerobic capacity was reassessed. Biopsies were collected pre and post endurance training with Bergstroem needles, frozen in nitrogen-cooled isopentane and stored in liquid nitrogen until further analysis was performed.

### Muscle structure

Quantitative alterations in muscle ultrastructure were evaluated with established morphometric technique from glutaraldehyde-fixed muscle biopsies (Schmutz et al. 2006). In brief, this comprised the assessment of volume densities of myofibrils, total mitochondria, subsarcolemmal mitochondria, intramyocellular lipids, residual organelles and capillaries. In addition, mean muscle fibre cross sectional area (CSA), capillary-to-fibre ratio and slow fibre type content were estimated (Suter et al. 1995).

### Transcript profiling

Level alterations of 231 gene transcripts were assessed in total RNA from a subset of bicycle-trained subjects prior to and during recovery from bicycle exercise in the untrained state and after bicycle training with validated low-density Atlas^®^ cDNA arrays (BD Biosciences, Allschwil, Switzerland; (Schmutz et al. 2006). The genes included on the array platform covered major gene ontologies (GO) underlying muscle energy and protein metabolism, fibre structure, cell regulation and angiogenesis (GPL 1935, Gene Omnibus: http://www.ncbi.nlm.nih.gov/geo). The selected time points (i.e. 1, 8 and 24 h) were chosen in reference to the peak response of transcript expression in untrained subjects (Schmutz et al. 2006). Microarray data series were deposited under accession codes GSE 13623 and GSE 2479, respectively, at Gene Omnibus.

### Statistical analysis

Data were organised in MS-Excel and exported into Statistica 9 (Statsoft) for statistical testing. Physiological and structural variables were assessed with a Student’s *t* test. A one-factorial ANOVA was carried out to assess the effect of carrying the ‘*ACE I*-allele’ (i.e. combined *ACE*-*II* and *ACE*-*ID* genotypes vs. the *ACE*-*DD* genotype) for the measured parameters (ultrastructural variables, relative and absolute *V*O_2max_, PPO). The interaction effect of the co-variable of ‘body weight’ and the ‘ACE I-allele’ on relative and absolute *V*O_2max_ was assessed with a general (non)linear model. A repeated ANOVA was used to compare ‘post versus pre’ effects of exercise or training and its interaction with the ‘*ACE I*-allele’. For the latter test, the data were normalized to the mean value for the respective genotype prior to training. The calculated *p* values (α = 0.05 except stated otherwise) of the post hoc tests were adjusted for the error of testing multiple, independent hypotheses (*m*) by applying a Bonferroni correction (i.e. setting the new *p* value to *α/m*). Effects were called significant at the Bonferroni-corrected *p*-value when *p* < α*/m*.

The evaluation of the gene expression data was as follows: Microarray data were background corrected as described (Schmutz et al. 2010). Subsequently, background-corrected signals were related to the total count of transcript signal per array to reveal normalized values. Significant adjustments in muscle transcript expression following a single bout of endurance exercise and training between carriers and non-carriers of the *ACE I*-allele were evaluated from normalized values using a Significance Analysis of Microarrays test (SAM) running as an applet in Microsoft Excel software (Tusher et al. 2001). Default settings for two-class, paired data with post hoc *t* tests were used during the calculation. A *q* value being adjusted for the number of assessed gene transcripts (i.e. 5 %/231) was deemed appropriate to identify differently expressed gene transcripts.

Visualization of fold differences in transcript expression was achieved through treatment of the data with Cluster and Treeview software (http://rana.lbl.gov/EisenSoftware.htm). Postscript files were imported into CorelDraw X3 (Corel Corp.) and assembled in Powerpoint (MS-Office for Windows XP, Kildare, Ireland). Genmaps were constructed using Genmapp (www.genmapp.org).

## Results

### Differences in aerobic capacity between carriers and non-carriers of the *ACE I*-allele

**Fig. 1:**
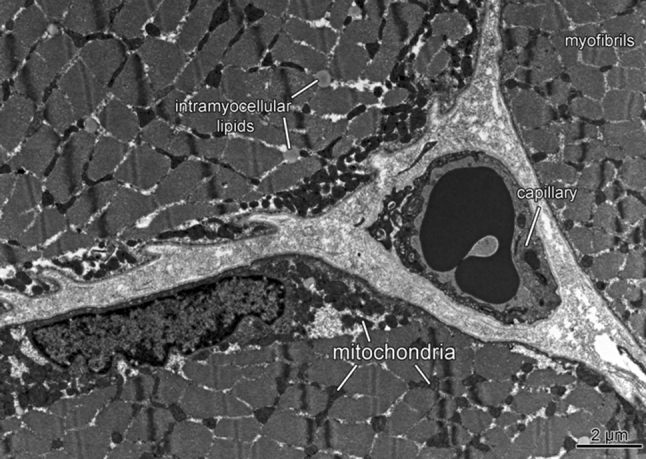
ACE I-allele dependent effects on muscle-related parameters of fitness. Representative micrograph indicating the assessed ultra-structural parameters in *vastus lateralis* muscle

At baseline, relative, but not absolute, *V*O_2max_ was lower in carriers than non-carriers of the *ACE I*-allele (Table 1a). No interaction effect was evident between the *ACE I*-allele and the co-variable of ‘body weight’ for absolute and relative *V*O_2max_ (*p* > 0.69). At the muscle level (Fig. 1), capillary density in *vastus lateralis* muscle was lower in carriers of the *ACE I*-allele.

**Table 1:** Differences in whole body (a) and muscle parameters (b) between carriers of the ACE I-allele (i.e. combined ACE-ID and ACE-II genotypes) and non-carriers (i.e. ACE-DD genotype) in the studied Swiss men before endurance training

Genotype	Non-carriers	Carriers	*p* value
a)			
Age (years)	29.9 ± 2.2	33.7 ± 1.9	*0.21*
Height (m)	178.2 ± 1.9	177.2 ± 1.7	*0.72*
Body weight (kg)	73.2 ± 2.2	80.2 ± 3.5	*0.14*
BMI (kg m^-2^)	23.0 ± 0.4	25.5 ± 1.0	*0.05*
VO_2_max [L O_2_ min^-1^]	3.43 ± 0.17	3.06 ± 0.13	*0.10*
VO_2_max [mL O_2_ min^-1^ kg^-1^]	47.1 ± 2.3	39.4 ± 1.8	*0.01*
n	14	21	
b)			
Fibre CSA (μm^2^)	3408 ± 242	3586 ± 226	*0.60*
Slow fibre type (%)	50.8 ± 4.9	51.3 ± 2.4	*0.20*
Capillary-to-fibre ratio	1.8 ± 0.1	1.5 ± 0.1	*0.05*
Capillary density (mm^-2^)	540.4 ± 26.5	438.9 ± 16.8	*0.002*
Total mitochondria Vv (%)	5.1 ± 0.3	4.8 ± 0.3	*0.48*
Subs. mitochondria Vv (%)	0.8 ± 0.1	0.8 ± 0.1	*0.71*
Intramyocellular lipid Vv (%)	0.6 ± 0.1	0.6 ± 0.1	*0.94*
Residual Vv (%)	13.7 ± 0.8	12.3 ± 0.4	*0.08*
n	14	21	

Data refer to mean ± Standard error (SE). *P* values of two-tailed *T*-test passing a Bonferroni-corrected *p* value < 0.05 are underlined

*Vv* volume density, *subs.* subsarcolemmal

### The *ACE I*-allele modulates the muscle response to bicycle type endurance training

**Fig. 2:**
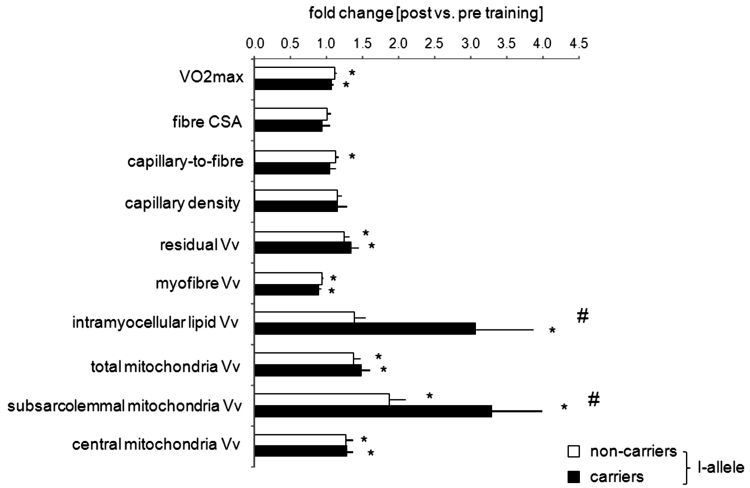
ACE I-allele dependent muscle adjustments to endurance training. Bar graph of mean + SE of fold changes in muscle parameters and VO_2_max in carriers and non-carriers of the ACE I-allele after bicycle training. *n* = 9 with no I-allele, 7 with I-allele. *Vv* volume density. *, *p* < Bonferroni-corrected 0.05 for post versus pre changes (paired *T*-test). # denotes a significant interaction effect between the fold-changes (‘post vs. pre-training’) and the ‘ACE I-allele’ at *p* < Bonferroni-corrected 0.05 (repeated ANOVA)

Absolute and relative VO_2_max was improved by 9 and 10 %, respectively, after bicycle type of endurance training (Table 2a). Bicycle training also increased the volume density of subsarcolemmal and total mitochondria and intramyocellular lipid in *vastus lateralis* muscle. The volume density of subsarcolemmal mitochondria and intramyocellular lipid demonstrated an interaction effect between the ‘*ACE I*-allele’ and ‘training’ (Fig. 2). Volume density of subsarcolemmal mitochondria was 3.3-fold and intramyocellular lipid was 3.1-fold elevated after bicycle training in carriers of the *ACE I*-allele. *ACE*-*DD* genotypes demonstrated a lower degree of volumetric increase in subsarcolemmal mitochondria and no change in intramyocellular lipid (Fig. 2).

**Table 2:** Adjustments induced in subjects which completed endurance training composed of 6-weeks of supervised bicycle exercise (*n* = 16, a) or 6-months of self-regulated running (*n* = 19, b)

Factor	Fold	*p*-value
a) bicycling		
Absolute VO_2_max	1.09 ± 0.02	2E−5
Relative VO_2_max	1.10 ± 0.01	2E−6
Body weight	0.99 ± 0.01	0.26
Fibre CSA	0.98 ± 0.05	0.76
Capillary-to-fibre ratio	1.10 ± 0.04	0.02
Capillary density	1.15 ± 0.06	0.01
Residual Vv	1.29 ± 0.05	9E−5***
Myofibre Vv	0.92 ± 0.01	8E−6***
Intramyocellular lipid Vv	2.13 ± 0.40	7E−4*
Total mitochondria Vv	1.43 ± 0.07	2E−6*
Subsarcolemmal mitochondria Vv	2.50 ± 0.36	1E−5**
Central mitochondria Vv	1.28 ± 0.06	8E−5
b) running		
Absolute VO_2_max	1.08 ± 0.02	3E−3
Relative VO_2_max	1.09 ± 0.03	3E−3
Body weight	1.00 + 0.04	0.20
Fibre CSA	1.03 ± 0.04	0.58
Capillary-to-fibre ratio	1.06 ± 0.04	0.15
Capillary density	1.05 ± 0.04	0.36
Residual Vv	0.99 ± 0.04	0.76
Myofibre Vv	0.99 ± 0.01	0.31
Intramyocellular lipid Vv	1.11 ± 0.18	0.49
Total mitochondria Vv	1.21 ± 0.06	4E−3
Subsarcolemmal mitochondria Vv	1.60 ± 0.23	0.07
Central mitochondria Vv	1.17 ± 0.06	0.01

*P*-values of paired *T* tests passing a Bonferroni correction are underlined

*Vv* volume density

*, ** and ***, respectively, denote *p*-values <0.05, <0.01 and <0.001 for the comparison of fold changes after bicycle versus running type endurance training

### *ACE I*-allele modulated local aerobic capacity depends on exercise type

Running type endurance training increased absolute and relative *V*O_2max_ by 8 and 9 %, respectively (Table 2b). As well, total mitochondrial density in *vastus lateralis* muscle was increased after running training and this distinguished to the effect of bicycle training (compare Table 2a, b). There was an interaction effect of ‘exercise type’ and ‘*ACE I*-allele’ for the fold changes of combined muscle parameters with endurance training (*p* = 0.024). No effect of the *ACE I*-allele on training-induced adjustments was observed in the running group (*p* values > 0.23).

### Exposing the molecular pathway of the *ACE*-dependent training response

**Fig. 3:**
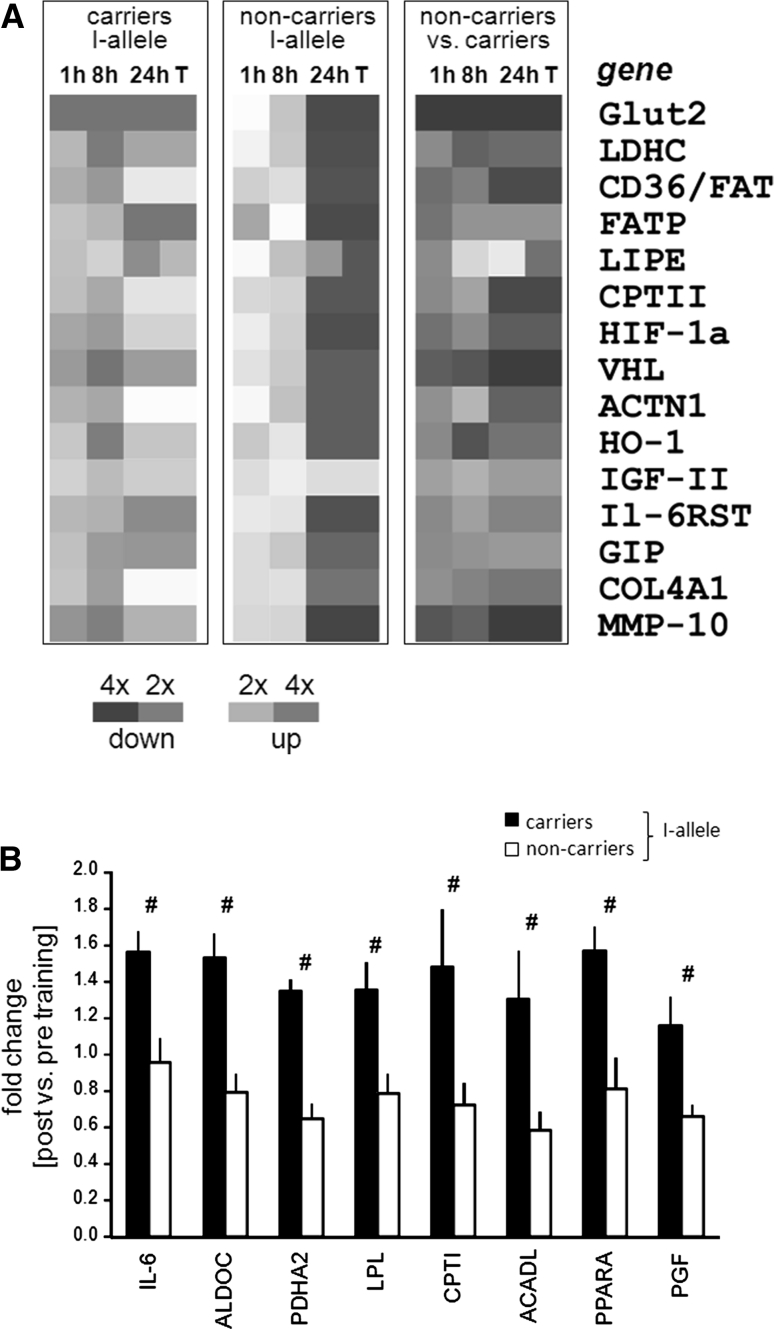
Genotype dependent expression changes to endurance exercise. **a** Heat map visualizing the regulation of the 15 gene transcripts and their functional ontology demonstrating level differences between carriers and non-carriers of the *ACE I*-allele in *vastus lateralis* muscle during recovery from bicycle exercise and after bicycle training. Colour code denotes the scale of fold changes 1, 8, 24 h after endurance exercise and training versus baseline in carriers of the I-allele, non-carriers of the I-allele, and the corresponding ratio between the two genotypes. **b** Bar graph of mean + SE for fold changes in training-induced expression for 8 transcripts which distinguished between ACE genotypes after training. #, *q*-value <5 %/231 for differences in fold changes between genotypes. *n* = 12. For abbreviations see legend to Fig. 4

24 of the 231 assessed muscle transcripts demonstrated level regulation after the first endurance exercise test and endurance training on the bicycle. The levels of 15 gene transcripts demonstrated *ACE I*-allele-dependent regulation during recovery from bicycle exercise and training (Fig. 3a). The majority of these were associated with glucose (i.e. Glut2, LDHC) and lipid metabolism (CPTII, LIPE, FATP, CD36/FAT) and metabolic regulation (HIF-1a, VHL, GIP, Il-6RST, IGF-II). 8 further gene transcripts associated with metabolic processes (i.e. ALDO C, CPTI, IL-6, ACADL, LPL, PDHA2, PPARA, PGF) demonstrated *ACE I*-allele dependent expression with training (Fig. 3b); all transcript changes with training showing a reduced magnitude in non-carriers of the *ACE I*-allele.

## Discussion

**Fig. 4:**
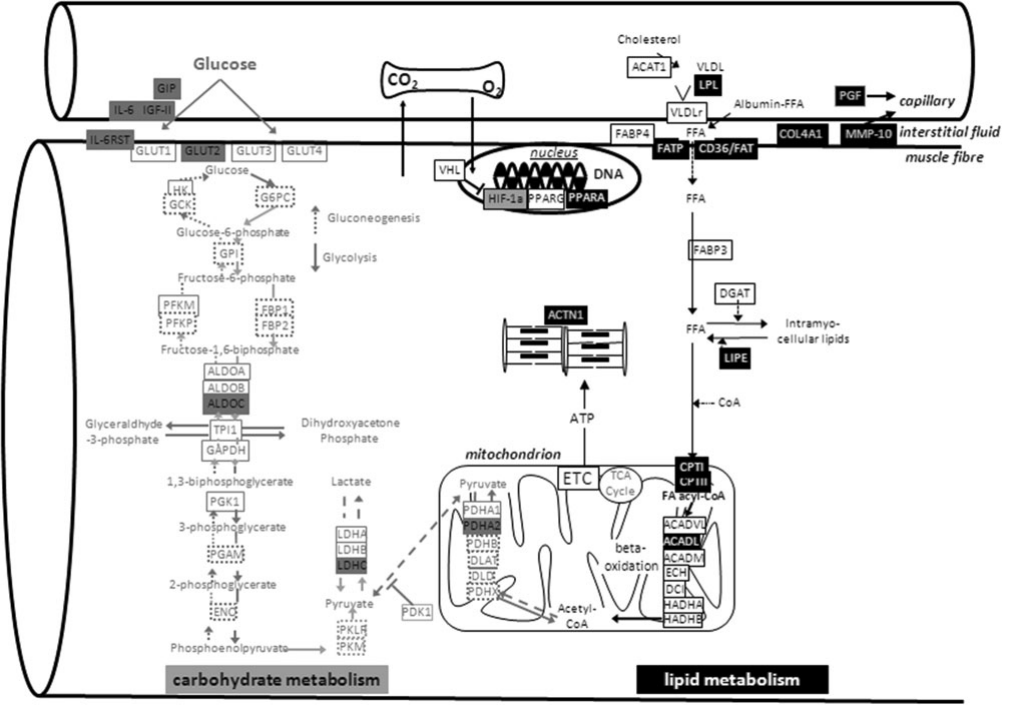
Summary of ACE I-allele affected pathways Genmapp visualizing the metabolic pathways holding gene transcripts with ACE I/D genotype dependent expression post exercise/training. The assessed transcripts are given in *boxes* with the *colour coding* indicting a significant genotype effect. *ACADL* long chain acyl-CoA dehydrogenase, *ACTN1* actinin alpha 1, *ALDOC* aldolase C, *COL4A1* collagen type IV alpha 1, *CPTI* carnitine palmitoyltransferase I, *CPTII* Carnitine palmitoyltransferase II, *CD36/FAT* cluster of Differentiation 36)/fatty acid translocase, *ETC*. electron transport chain, *FATP* fatty acid transport protein, *GIP* glucose-dependent insulinotropic peptide, *Glut2* Glucose transporter 2, *HIF-1a* subunit alpha of hypoxia-inducible factor 1, *HO-1* heme oxygenase 1, *IGF-II* insulin-like growth factor II, *IL-6* interleukin 6, *Il-6RST* interleukin 6 receptor signal transducer, *LDHC* lactate dehydrogenase C, *LIPE* hormone sensitive lipase transcript, *LPL* lipoprotein lipase, *MMP-10* metalloproteinase 10, *PDHA2* pyruvate dehydrogenase alpha 2, *PGF* placental growth factor, *PPARA* Peroxisome proliferator-activated receptor alpha, *VHL* von Hippel Landau tumour suppressor. For further abbreviations consult www.expasy.org

Supply of metabolic substrates into working muscle is an important component of endurance exercise capacity. Our present investigation shows that variants of the *ACE* insertion/deletion polymorphism fundamentally differ in muscle capillarity and the response of muscle mitochondria and intramyocellular lipid content to repeated endurance exercise. Transcript profiling exposed a number of genes associated with carbohydrate and lipid metabolism in skeletal muscle as targets of a novel, *ACE* modulated expression pathway being activated by endurance exercise (Fig. 4).

A number of limitations related to sample size for a genetic study, control of metabolite supply and protein verification of identified transcript differences, may be worth considering in the interpretation of our results. We note that the absence of a rigorous control for nutrient intake may produce uncertainty in our conclusions. Thus, our conclusions may be considered as preliminary as we cannot exclude that lifestyle associated factors have contributed to the observed effect of the *ACE* insertion allele on changes in intramyocellular lipid volume density after endurance training. Also, due to limitations in available amount of biological tissue, we were not able to assess whether expressional adjustments of proteins encoded by the genotype-affected transcripts (Fig. 4) underline the identified changes. Regarding the higher capillarity in the extensor muscle, *M. vastus lateralis*, of our Swiss *ACE*-*DD* genotypes before training, we find that this is consistent with the concomitantly elevated relative *V*O_2max_ (Table 1a) as expected from the contribution of oxygen delivery to extensor muscle groups during the dynamic knee extensor exercise of bicycling (Krustrup et al. 2004). This observation is in line with other Caucasian populations [reviewed by (Flueck et al. 2010)] while being at variance with early studies of Montgomery in trained British men [reviewed in (Puthucheary et al. 2011)]. In trying to address the role of exercise in potentially *ACE I*-allele modulated muscle plasticity, we assessed the effect of bicycle type endurance training on ultrastructure and transcript expression in *vastus lateralis* muscle. Because the association between endurance phenotypes and the *ACE I*-allele has been studied in endurance runners and cyclists (Alvarez et al. 2000; Myerson et al. 1999), we opted to compare the results of bicycle versus running type of endurance training. The amplified adjustments of myocellular organelles (Fig. 2) and gene transcripts being associated with mitochondrial lipid metabolism after supervised bicycle training (Fig. 3a) is in agreement with the hypothesis suggested from the literature on elevated trainability of endurance performance in subjects carrying the *ACE I*-allele (Myerson et al. 1999). Thus, our observations allow to draw a number of mechanistically important conclusions on the contribution of skeletal muscle to *ACE* modulated exercise performance and its molecular underpinning.

The higher capillary-to-fibre ratio and capillary density in *vastus lateralis* muscle in untrained participants with the *ACE*-*DD* genotype (Table 1b) exposed that the ACE I/D genotype modifies muscle quality by an effect on angiogenesis. It has been demonstrated that the product(s) of the *ACE* gene product, angiotensin 2 and bradykinin, are potent stimulators of angiogenesis in model systems (Amaral et al. 2001; Heffelfinger 2007; Petersen and Greene 2007). In this regard, it is of note that non-carriers of the *ACE*-*I* allele demonstrate elevated ACE activity (Alvarez et al. 2000; Danser et al. 2007; Rigat et al. 1990). Our present novel observation indicates that genetic regulation of ACE affects in addition to the acute regulation of vascular tone (Santana et al. 2011) also the structural aspects of muscle perfusion.

In contrast to our hypothesis, muscle capillarity was not differently affected between carriers and non-carriers of the *ACE I*-allele with bicycle type or running type endurance exercise (Fig. 2, data not shown). The detailed inspection of the transcript response after bicycling identified, however, two factors being associated with angiogenesis (i.e. PGF and MMP-10) whose expression in exercised muscle was affected by the *ACE I*-allele (Fig. 3a). Adjustments in muscle capillarity with endurance training are known to grade to exercise intensity and manifest predominantly after high intensity protocols of training (Egginton et al. 2001; Jensen et al. 2004; Lampert et al. 1998). Combined, these findings suggest that the investigated endurance protocols did not create the biological context, to resolve whether an exercise-mediated mechanism contributes to *ACE* modulated capillarity.

Conversely, we observe a specifically enhanced improvement in volume density of subsarcolemmal mitochondria and intramyocellular lipid in *vastus lateralis* muscle with bicycle training in carriers of the *ACE I*-allele (Fig. 2). This finding points towards a role for the *ACE I/D* polymorphism in regulating local adjustments in aerobic metabolism in a major extensor muscle. The concomitantly amplified up-regulation of transcripts for factors of lipid and carbohydrate metabolism in carriers of the *ACE I*-allele during recovery from endurance exercise and training (Fig. 4) identifies that a novel *ACE I*-allele modulated expression pathway contributes to the conditioning of muscle’s aerobic capacity in men. In this context it is of interest that carriers of the *ACE I*-allele demonstrated reduced muscle capillarity and maximal oxygen uptake at baseline (Table 1b). The observations in our population support the hypothesis that remodelling of aerobic substrate pathways in skeletal muscle contributes to the reported superior trainability of endurance performance in carriers of the *ACE I*-allele (Cam et al. 2007; Flueck et al. 2010).

A specific observation of our study was that the ultrastructural adjustments in the knee extensor muscle, *vastus lateralis*, differed between bicycle and running type endurance exercise despite a similar increase in maximal oxygen uptake (Table 2). This is in line with the pronounced effects of the employed bicycle paradigm on mitochondrial volume density in *vastus lateralis* muscle (Hoppeler et al. 1985; Hoppeler and Weibel 1998). It possibly reflects a more important contribution of the investigated extensor muscle to power output during a bicycle than running type workout. Possibly, the self-controlled nature of the running training, despite being verified for compliance, contributed to the observed differences in adaptations between the two types of endurance training, as well. The superior myocellular adjustments in the volume density of subsarcolemmal mitochondrial and intramyocellular lipid stores after bicycle type compared to running type endurance training, and their dependence on the *ACE I*-allele is in agreement with the idea that the association between the *ACE I*-allele polymorphism and endurance performance is intensity dependent (Cam et al. 2007). Our findings now point out that the exercise type is a potential confounding variable when interpreting results from genetical studies into human performance.

In contrast to elevated subsarcolemmal mitochondria density in carriers of the *ACE I*-allele after bicycle training, the improvements in absolute and relative VO_2_max were not significantly affected by the *ACE I/D* polymorphism (Fig. 2). This suggests that the *ACE* genotype may influence aspects of mitochondrial metabolism that do not necessary manifest in elevated aerobic capacity. In this regard, it is of interest that subsarcolemmal mitochondria relate to a better use of blood-borne substrate following endurance exercise [reviewed in (Lampert et al. 1998)] as they situate in vicinity to capillaries (Fig. 1). This view is supported by the specific higher up-regulation of transcripts involved in mitochondrial beta oxidation (CPTI, CPTII, ACADL), but not the electron transport chain after training in carriers compared to non-carriers of the *ACE I*-allele (Fig. 4).

In support of modified metabolism in exercised muscle between genotypes, we identify a selective increase in intramyocellular lipid density after endurance training in carriers of the *ACE I*-allele. Our observation represents to the best of our knowledge the first documentation of ACE-related muscle lipid content in healthy humans after training. Interestingly, exercise-induced hypotension, reflecting elevated muscle perfusion, is confined to carriers of the *ACE I*-allele (Santana et al. 2011). Muscle perfusion likely affects intramyocellular lipid stores as these arise through the esterification of fatty acids taken up from the vasculature into perfused muscle during recovery from exercise and synthesis from long-chain fatty acids (Jayakumar et al. 1995; Kiens 2006). In this regard, it is of note that transcript profiling identified pronounced *ACE I*-allele dependent up-regulation of two transcripts involved in fatty acid mobilisation (i.e. CD36/FAT, FATP) during the first 24 h of recovery from exercise and after training (Fig. 3a). As well, the LPL transcript for lipoprotein lipase was selectively increased in carriers of the ACE I-allele after bicycle training (Fig. 3b). FATP (fatty acid transport protein) and CD36/FAT (fatty acid translocase) and LPL encode factors being involved in the uptake of free fatty acids in muscle fibers during recovery from exercise (Bonen et al. 2004; Kiens 2006). The findings indicate a role of the *ACE I/D* polymorphism in an expressional mechanism setting the improvement in uptake capacity for free fatty acids into skeletal muscle and intramyocellular lipid accumulation after repeated endurance exercise stimulus. The content of intramyocellular lipid relies, however, on multiple biochemical reactions. In this regard, it is of note that the LIPE transcript levels for the suspected major lipase for hydrolysis of stored triacylglycerol in skeletal muscle showed ACE-genotype modulated regulation after endurance training (Fig. 3a). This emphasises that multifactorial expressional mechanism possibly mediates the indicated effects of the *ACE I*-allele on lipid metabolism in skeletal muscle.

Support for a broader influence of the ACE I/D polymorphism on metabolic regulation is provided by elevated muscle transcript levels of transcriptional regulators of lipid (i.e. PPARA) and carbohydrate/mitochondrial metabolism (i.e. HIF-1a, VHL) and three glycolytic factors after single or repeated exercise in carriers of the *ACE I*-allele (Fig. 4). HIF-1a is the hypoxia-regulated subunit of the hypoxia-inducible transcription factor HIF-1 that is stabilized by VHL. Increased levels of the HIF-1a transcript promote mitochondrial and glycolysis related transcript expression in mouse skeletal muscle (Däpp et al. 2006), the human homologues of five of which (i.e. ACADL, Glut2, LDHC, LPL) demonstrated ACE I-allele related expression after bicycle endurance training (Fig. 4). Given that the *ACE*-*DD* genotypes of our study group show elevated capillarity before, and after, endurance training (Table 1b, data not shown) this bears the question whether the promoted expression of metabolic features after bicycle training in carriers of the *ACE I*-allele reflects a strategy to compensate enhanced local metabolic strain during endurance exercise (reviewed in Schmutz et al. 2010).

## Conclusions

Our findings indicate that *ACE I*-allele modulated capillary supply lines affect adjustments in mitochondrial lipid metabolism in skeletal muscle in relation to altered transcript expression after intense endurance exercise. *ACE I*-allele modulated metabolic gene expression is suggested as a partial explanation for the superior response of endurance capacity to endurance training in human subjects with the *ACE I*-allele.
